# Growth of prepubertal children on dialysis

**DOI:** 10.1007/s00467-007-0481-7

**Published:** 2007-09-01

**Authors:** Constantinos J. Stefanidis, Günter Klaus

**Affiliations:** 1 Department of Nephrology, “P. & A. Kyriakou” Children’s Hospital of Athens, Goudi, 14562 Athens, Greece; 2 Department of Paediatrics, University of Marburg, Marburg, Germany

**Keywords:** Chronic kidney disease, Dialysis, Growth failure, Malnutrition, Renal bone disease, Recombinant human growth hormone

## Abstract

Growth failure is a common and significant clinical problem for children on dialysis and often remains a major impediment to their rehabilitation. Early referral to a paediatric nephrology centre and appropriate management before the initiation of dialysis may significantly prevent growth deterioration. Growth in children on dialysis can be affected by nutritional, metabolic, and hormonal changes. Early diagnosis of malnutrition and aggressive management should be a priority. Gastrostomy feeding should be used when adequate oral intake to maintain normal height and weight velocity cannot be achieved. Active vitamin D metabolites should be used carefully, to prevent low-turnover bone disease. All children should have an adequate regimen of dialysis and an appropriate management of malnutrition, renal osteodystrophy, metabolic acidosis, salt wasting and anaemia, before recombinant human growth hormone (rhGH) administration is considered. The current challenge of reversing growth impairment in children on dialysis can only be achieved by optimization of their care.

## Introduction

It has long been recognized that growth retardation is a common complication of children on dialysis [1, 2]. This problem remains a concern today, despite the radical improvements in their management over the past two decades. This concern has recently increased, because poor growth of children with chronic kidney disease (CKD) has been associated with increased risk of hospitalization and death [3]. The most common causes of hospitalization in these patients are infections, possibly related to their poor nutritional status. Therefore, although poor growth is unlikely to be the cause of this increased morbidity, growth failure may be a marker of untoward events [3]. The decrease of height velocity before and during the dialysis period frequently results in diminished final adult height [4]. Children with extremely short stature face a disability that may affect their psychological and social well-being [5]. In this issue of *Pediatric Nephrology* Cansick and co-workers report on the growth of 35 prepubertal children who have been on dialysis for more than 1 year [6]. The results of this retrospective study are encouraging, since the majority of patients had no deterioration of their growth. In addition, catch-up growth was observed in some children under 2 years of age within the first dialysis year. This was achieved with intensive medical and nutritional therapy. Ninety-one percent of patients received supplementary enteral feeding, either by nasogastric tube or by gastrostomy tube for some or all of the study period. In addition, all patients had meticulous management of their renal osteodystrophy, and the median parathyroid hormone (PTH) level was 1.5-times the upper limit of normal [6]. These excellent results were accomplished without the daily administration of recombinant human growth hormone (rhGH).

Growth retardation in children on dialysis is the consequence of a combination of several inter-related processes, which are partially understood. The major factors responsible include malnutrition, metabolic bone disease, anaemia, salt-wasting disorders, metabolic acidosis, reduced responsiveness to endogenous growth hormone (GH) and insulin-like growth factor (IGF)-I and other endocrine abnormalities [7]. The positive effect of adequate dialysis on growth is also well documented in patients on peritoneal dialysis (PD) [8, 9]. A subset of children on dialysis may still have poor linear growth, despite the optimal management of all the treatable factors. For these patients the administration of rhGH is recommended.

### Management of growth retardation before the initiation of dialysis

Data on growth of 2,329 children in the North American Pediatric Renal Transplant Cooperative Study (NAPRTCS) revealed that the mean height standard deviation score (SDS) at the initiation of dialysis was −2.54, −1.95, and −1.67 for children aged 0 to 1 years, 2 to 5 years and 6 to 12 years, respectively [10]. The results of the study by Cansick et al. were similar: the mean SDS at the start of dialysis was −2.1 [6]. This means that 50% of the prepubertal children of this study had poor growth at the initiation of dialysis, despite the adequate nutritional care that is provided in this centre. Those findings suggest that the strategies for growth improvement before dialysis were suboptimal and should definitely be improved. In addition, this shows that rhGH before the initiation of dialysis is not widely used. Late referral to a paediatric nephrology centre might partially explain these findings. Several recent studies on adults have documented that late referral may significantly contribute to significant complications with dialysis [11–13]. A recent multicentre study from Poland found that 21% of 180 children started dialysis within 1 month after their first referral to a nephrologist and were classified as late referrals. These children had a body mass index below the 10th percentile significantly more often than children with early referral [14]. Early referral followed by appropriate nutritional management may lead to prevention of growth deterioration. However, if conservative management is not effective, rhGH should be administered before the initiation of dialysis. Short children on conservative management respond better to rhGH than do children on dialysis [15]. Early referral may also result in early initiation of dialysis and preservation of residual renal function (RRF). There is evidence that RRF has a positive effect on growth of children on PD [8].

### The effect of dialysis modality on growth

The initiation of haemodialysis (HD) usually does not improve growth [1]. Actually, a mean of 0.4–0.8 height SD was lost per year in various studies [1, 16]. Recent data from the Annual Report of NAPRTCS are more optimistic. [10]. It is of interest that an annual height gain of 0.3 SD was observed in 12 children on long-term HD treated with a combination of adequate nutrition and with dialysis urea clearances exceeding conventional recommendations (mean Kt/V_urea_ of 2, and average time of HD of 15 h/week) [17]. Studies on adults show superior dialysis adequacy on slow nocturnal HD compared with conventional HD. It is expected that this technique will have a positive effect on growth. Presently, there are no data on growth of children on slow nocturnal HD. However, a recent preliminary report suggests the potential efficacy of in-centre intensified and daily HD, delivered five to six times weekly, for 3 h each session. Five children treated with this regimen had a mean change of +1.84 height SDS/year [18].

Despite some optimistic early reports on growth of children on PD [2, 19], more recent studies confirm percentile-parallel growth patterns in the majority of patients. Changes from baseline of mean height SDS were −0.04 (*n* = 1,320) and −0.09 (*n* = 525) at 12 months and 24 months of PD, respectively, at the 2006 Annual Report of NAPRTCS. For children on HD, these changes were −0.14 (*n* = 573) and −0.43 (*n* = 276) at 12 months and 24 months [10]. The majority of children at initiation of PD are younger than patients on HD, and this selection might explain the above differences in growth. There is evidence that growth of children on dialysis is better during the first 2 years of life. This has been confirmed by the study by Cansick et al. [6]. In addition, changes from baseline of mean height SDS of patients less than 1 year were 0.27 (*n* = 263) and 0.40 (*n* = 100) at 12 months and 24 months of dialysis in the report of NAPRTCS [10]. The growth data of 51 children and adolescents on PD from 16 mid-European paediatric dialysis centres were similar to those of NAPRTCS. The mean height (SDS) was −2.0 at the start of PD. During the 18-month study period, the degree of growth retardation did not change significantly (mean height SDS −2.1) [20]. The risk of losing height SDS was increased in high transporters, who exhibited an average loss of 0.5 SD units per year of dialysis [20]. Dialytic creatinine clearance and total dialysate volume were significant predictors of the change in height SDS in these patients [20]. Therefore, optimal prescription of volume should be a priority for all children on PD, and this can be achieved with the adherence to guidelines for the paediatric peritoneal dialysis prescription [21]. In children, a Kt/V_urea_ over 2 has to be achieved, but this may be difficult in those who are anephric or have minimal residual function [21]. Better growth results in children on PD are described in single-centre studies. Catch-up growth occurred in 62% of 21 children on PD in a study from Helsinki [22]. The primary renal disease of the children under 5 years of age was mainly congenital nephrotic syndrome, with mutation in the nephrin gene (*NPHS1*). The 9-month change in height SDS in patients under 5 years of age was significantly better in patients under 5 years of age than in the older patients [22]. Despite the fact that only one patient received rhGH, the mean height SDS of the group increased from −1.3 ± 1.2 at baseline to −0.8 ± 1.0 at 9 months. This was probably the result of augmented weekly mean solute clearances (Kt/V_urea_: 3.2 ± 0.6 and creatinine clearance 69 ± 17 l/1.73 m^2^ body surface area per week) [22]. Similarly, McCauley et al. reported catch-up growth in nine of 15 children treated with PD [23].

There is evidence for a positive influence of residual renal function on the growth of children receiving PD [8, 24]. Catch-up growth was observed in 58% of children with RRF, but such growth was seen only in 17% of 12 patients without RRF [8]. It is of interest that patients with and without RRF had near-equal total Kt/V_urea_ values. These findings challenged the concept of the equivalence of solute removal by native kidney function and by dialysis and documented the superiority of RRF. Better preservation of RRF with PD than with HD was reported in adult [25] and paediatric [26] patients. It has been recently reported that RRF appears to play a greater role in paediatric patients on HD than was previously recognized, by improving dietary prescription and hypertension [27]. The maintenance of RRF also has implications for morbidity in adult dialysis patients by causing better dialysis clearance, elimination of middle molecules and β2-microglobulin, and improved fluid–electrolyte balance and cardiovascular morbidity [28].

Recently, an improvement in the growth of children on dialysis was reported. In 1987, patients receiving their initial transplant were an average of 2.4 standard deviations below average [10]. This improved to −1.6 in the 2005 cohort of NAPRTCS. However, in the same cohort, the average height SDS was −2.6 for children 2–5 years of age [10]. In addition, the weakness of the NAPRTCS report is the fact that all the analyses regarding growth are based on cross-sectional data and not on longitudinal data. For this reason, the number of patients is changing at the time intervals of the report. The comparison of the cross-sectional data from 1987 to cross-sectional data differently collected in 2006 is a weakness of the study, and this is the major criticism of the optimistic information of this report.

## Assessment of malnutrition

Caloric deficiency and abnormal protein metabolism may play an important role in growth impairment, particularly in younger children. This problem is common in children on dialysis [29, 30] and is usually mild [31, 32]. Malnutrition in our day is less often noted than in the past; however, it is still seen in many centres. There is a lack of generally accepted criteria for the diagnosis of this problem. In addition, there is a problem with the interpretation of weight (and body mass index) in dialysis patients because of the difficulties in estimating their true dry weight. Bio-electrical impedance (BIA) is a promising technique for the assessment of the state of hydration [31, 33]. The lack of invasiveness, and the low cost of this method, make it very attractive. In addition, with this technique, it is easy to calculate whole-body percentage fat-free mass (FFM) [34]. These calculations are based on the assumption that the percentage of water of the FFM is stable (73%). However, in children on dialysis, this assumption is not always true, and, therefore, FFM prediction might be extremely problematic. Mid-upper arm circumference and skinfold thickness measurements from many sites are widely used for estimating body composition [35]. Despite the limited precision of these measurements, acceptable reproducibility can be achieved with skinfold thickness measurements, and these are useful for monitoring changes over time. Recently, a nutritional score was developed, based on the following three groups of parameters: anthropometry 1 (height, weight, body mass index), anthropometry 2 (mid-arm muscle circumference, arm muscle area, and arm fat area) and BIA (reactance, phase angle, and distance) [31]. Each parameter received a score of 5 for values of > 0 SDS; 4 for values of 0 and > −1 SDS; 3 for values of −1 and > −2 SDS; 2 for values of −2 and > −3 SDS; and 1 for values of −3 SDS. An average score was calculated for each of the three groups, and these were added to obtain the anthropometry-BIA nutrition (ABN) score, which could vary from 3 (worst) to 15 (best). The ABN score corresponding to the 3rd percentile in the population of healthy children was 10.3. Therefore, the patients with ABN scores of < 10.3 were classified as malnourished. It is of interest that, when using this score, a recent Italian multicentre study found that 48% of children on PD were malnourished [31]. However, the percentage of children with scores suggesting severe malnutrition (ABN score of < 6) was very low (2.3%), whereas moderate (ABN scores of 6–8) and mild (ABN score of 8–10.3) malnutrition were equally distributed. This ABN score is a simple method for the assessment of the nutritional status and might prove to be very useful for identifying children with mild and moderate malnutrition [31].

Serum albumin concentration was frequently used as a reliable biochemical marker for nutritional status. However, the presence of acute or chronic inflammation limits the specificity of serum albumin as a nutritional marker [35]. Low values of serum albumin are closely related to the presence of inflammation. Serum albumin has been identified as a surrogate marker for morbidity/mortality in adult dialysis patients. It is of interest that paediatric patients who are started on dialysis when they exhibit hypo-albuminaemia are also at a higher risk of death [36]. Therefore, it is of concern that the prevalence rate of hypo-albuminaemia (serum albumin of < 2.9 g/dl) in children on PD was higher than that of adults (35.9% versus 19.5%; *P* < 0.004) [37]. Hypo-albuminaemia in children on PD was associated with a low protein catabolic rate, which was indicative of insufficient protein intake for their obligate protein losses during PD. This inadequate protein intake was considered responsible for the poor growth of patients [37].

### Management of malnutrition

Children on dialysis are often anorectic. Poor energy intake is the result of many factors, including psychological disturbances. Provision of sufficient energy is considered the most important factor in preventing poor growth in children on dialysis. The negative effect of malnutrition on growth during the first 2 years of life is well documented [38, 39]. Adequate provision of energy intake in infancy and early childhood can lead to catch-up growth [40]. However, a similar effect was not usually observed in older children [41], possibly because the nutritional management of these patients was not optimal. The initial prescribed energy intake for children treated with maintenance HD or peritoneal dialysis should be at the recommended dietary allowance (RDA) level for chronological age, since there is no evidence that children on dialysis require a higher RDA than do healthy children. Modifications should then be made depending upon the child’s response [35]. Children on dialysis should have their initial dietary protein intake based on the RDA for chronological age, with an additional increment of 0.4 g/kg per day for patients on HD, or their anticipated peritoneal losses for children on PD [35]. In many young children these goals can be accomplished only by supplementary enteral feeding. Of 101 children with severe chronic renal failure, presenting within the first 6 months of life at the Great Ormond Street Hospital for Sick Children in London, 81% were enterally fed for a median of 1.9 years. Catch-up growth occurred in many patients in the study [40]. Nasogastric tube feeding has been the most frequently used approach for nutritional support in children on PD [42]. However, with this technique, gastro-oesophageal reflux and aspiration remains a concern [43]. Eating difficulties following the withdrawal of nasogastric tube feeding is also an important problem, especially when it is initiated during the first year of life [44]. To avoid these problems, many centres prefer the use of gastrostomy feeding [45, 46]. In children on PD a gastrostomy should be created as on open procedure, where the stomach is directly connected to the anterior abdominal wall, with an initial Foley balloon catheter and subsequent insertion of a gastrostomy button [43]. The alternative approach to creating a gastrostomy is to use percutaneous endoscopic gastrostomy (PEG) [47]. However, this technique is frequently associated with the development of peritonitis in PD patients and should be used before the initiation of PD. It was recently reported that ten of 27 of children (37%) on PD developed peritonitis within 7 days of PEG insertion, and fungal peritonitis occurred in seven of 27 (26%) [47].

It is of interest that the requirements for energy intake was 125% of that recommended to achieve catch-up growth [48]. Although sufficient energy intake is usually recommended for children with growth retardation, an exaggerated calorie intake should be avoided [49]. An excessive energy intake may induce hyperlipidaemia and hyperinsulinism [49]. It is also associated with high leptin levels, which may contribute to lack of appetite [50]. Therefore, body mass index should be regularly calculated and must remain within the normal limits. An excessive protein intake should also be avoided in children on dialysis, to prevent metabolic acidosis, hyperphosphataemia and the accumulation of toxic nitrogen waste products.

### Renal bone disease and growth

Childhood renal osteodystrophy (ROD) is the consequence of disturbances of the calcium-regulating hormones vitamin D and PTH [51], as well as of the somatotroph hormone axis associated with local modulation of bone and growth cartilage function [52]. Although gross symptoms of renal osteodystrophy (bone deformities) are usually not noted in children with glomerular filtration rate (GFR) > 50 ml/min per 1.73 m^2^ body surface area, histological abnormalities of bone formation can already be noted in up to 50% of untreated patients [53]. Renal osteodystrophy affects growth not only as a consequence of gross bone deformities, but also by more subtle changes in growth plate cartilage function and in bone formation. Experimental data demonstrate several alteration of the growth plate and/or growth plate chondrocyte function. Both the severity and duration of renal failure may affect the growth plate differently [54]. Both the activity of the growth plate cartilage, by altering chondrocyte hypertrophy, and the replacement of cartilage by bone at the metaphyseal end are affected. The two processes are differentially depressed, since cartilage resorption is more severely lowered than is cartilage enlargement, and this leads to an accumulation of cartilage at the hypertrophic zone [55]. Therefore, growth plate proliferation as well as differentiation are affected by the uraemic state. A decreased immunohistochemical detection of growth hormone receptor [56] and insulin-like growth factor-I mRNA [57] in the proliferative zone of the uraemic growth plate was reported. The following sections concentrate on the effects of vitamin D and PTH on growth.

### The role of vitamin D

Already by the 1980s, Chesney et al. had documented the effect of calcitriol in preventing severe bone deformities in children with chronic renal failure (CRF) and had shown catch-up growth [51]. In experimental models catch-up growth was also observed in uraemic animals treated with vitamin D or active vitamin D metabolites. However, the improved growth could not solely be contributed to the effects of vitamin D alone [58]. In addition, this positive effect on growth could not be sustained for long periods. Supplementation with vitamin D_3_ and active vitamin D metabolites is essential, not only to regulate PTH production, but also for normal growth plate cartilage growth and differentiation in vitro [59, 60] and in vivo [61, 62]. However, high doses of calcitriol were shown to inhibit cartilage proliferation in vitro [59, 60] and in vivo [61, 62]. Calcitriol exerts a biphasic effect on chondrocyte proliferation in vitro. Low concentrations were shown to stimulate growth plate chondrocyte proliferation, whereas high concentrations showed an inhibitory effect in vitro [59, 60]. In animal experiments, intermittent high doses of calcitriol inhibited longitudinal growth and even abrogated the effect of growth hormone [62, 63]. Reduced growth from intermittent high doses of calcitriol was also reported in children on PD [64], but not in a controlled study in prepubertal children with pre-terminal renal failure in whom PTH was maintained at 2–3 times the upper limit of normal [65]. In children as well as in adult patients high-dose intermittent calcitriol regimens might result in suppressed bone formation (adynamic bone disease).

### Role of parathyroid hormone

Parathyroid hormone acts on the growth plate and on bone. In uraemia the amount of PTH/PTH-related protein receptor mRNA in bone cells [66] and in the animal growth plate [61, 67, 68] was found to be reduced, indicating a peripheral resistance to the action of PTH. Therefore, higher PTH concentrations were advocated for patients with CRF. Adynamic bone lesions were reported with PTH levels up to three-times the upper limit of normal [69]. When PTH serum levels are suppressed to normal values by active vitamin D metabolites, the risk of hypercalcaemia is increased, due to the inability of bone to incorporate calcium [70]. The guidelines of the Kidney Disease Outcomes Quality Initiative (KDOQI), as well as the recommendations by the European Pediatric Dialysis Working Group (EPDWG), recommend PTH levels within normal range in CKD stages 2–3 and up to three-times normal in stage 5 [71, 72]. It is not known which PTH levels should be aimed at in children with stage 4 CKD. Higher levels of PTH are often associated with hyperparathyroid bone disease. Marked hyperparathyroidism was associated with a trend to reduced growth in the retrospective study by Waller et al. [73]. Stunting of growth in marked hyperparathyroidism could be due to altered growth cartilage function, as demonstrated in animal [61] and in vitro [60] experiments. Osteopenia was also increased in children with CRF with high PTH levels (> 200 pmol/l) [74]. Bone mineral density was positively correlated with calcium and negatively with PTH and phosphorus serum levels [74]. In healthy adolescent girls PTH correlated inversely with bone mineral content [75]. In concordance with these guidelines, Waller et al. documented catch-up growth using a strict regime of phosphorus and calcium control in combination with enteral tube feeding and maintenance of PTH within the normal upper range in children with CKD stages 2–4 [73]. The same group report in this issue of the Journal [6] that there was no deterioration of growth of children on dialysis when PTH levels remained within 1.5-times the upper limit of normal. Children on dialysis who were below the (height)–age of 2 years even showed catch-up growth. This is in line with the physiological concept that longitudinal growth is mainly dependent on adequate caloric and protein intake within the first 2 years. In older children the action of growth hormone overrides the nutritional effect.

### Biochemical markers

The validity of biochemical assessment of bone turnover in advanced renal failure is still being debated. There are several markers of bone formation and bone resorption in adults [76, 77] as well as in children with CKD [78, 79]. The disadvantage of most markers is their low sensitivity and specificity to discriminate between the main types of renal bone disease, i.e. low-turnover (adynamic), normal and high-turnover bone formation. In adult dialysis patients, total alkaline phosphatase, as well as bone alkaline phosphates, predicted normal bone formation rate and adynamic bone disease taken as a group together versus high-turnover bone disease, with a sensitivity of 76% and specificity of 60% [77]. Bayazit and co-workers [79], as well as the group of Avila-Diaz [80], reported a correlation of PTH and alkaline phosphatase in children on dialysis. In the latter group, patients with PTH of less than 150 pg/ml were classified as low bone turnover patients. In these patients, alkaline phosphatase did not correlate with growth parameters. However, a positive correlation of alkaline phosphatase, but not of PTH serum levels, with growth was reported in the paper by Cansick et al. [6]. In another study a significant correlation was found between bone-specific alkaline phosphatase, as well as total alkaline phosphatase, and growth velocity in the preceding 6 months in children with CKD [81]. Possibly, this was the result of the absence of significant adynamic or high turnover bone disease, since there is a correlation between alkaline phosphatase and bone formation rate. This was achieved with the careful use of active vitamin D metabolites, which were shown to increase the incidence of low-turnover bone disease. It is well documented that low-turnover bone disease, as well as high-turnover bone disease (osteitis fibrosa), are associated with reduced growth. Cansick et al. report, in this issue of the Journal, a trend of reduced growth (though not significant) in the group of children with grossly elevated PTH serum levels compared with those with PTH up to three-times the upper limit of normal. However, owing to the retrospective design of the study, there was no histological evaluation of the bone.

### Treatment of renal growth failure with recombinant growth hormone

During the first 2 years of life, nutrition is the most important factor for growth. However, during childhood, the role of the somatotropic hormone axis becomes more important. The growth pattern during this period is usually parallel to the percentile curves, and it has been interpreted as “normal” growth, but this is underestimating the growth problem [7]. The standard deviation of height increases with age, and a male child with a height SDS of −3 in his 1st year of life has a height 7.6 cm below the median for his age and gender. This deficit will increase to 12.5 cm and 16.5 cm for this patient if his height SDS remains the same (−3) at the age of 4 years and 8 years, respectively [7]. Therefore, early management of growth retardation is crucial to avoid permanent reduction of height, and rhGH administration should be considered in the cases where nutritional intervention is not successful. The current guidelines recommend that rhGH be administered to children with CKD who have a height or height velocity for chronological age of below −2 SDS [34]. These guidelines also recommend that rhGH be prescribed once it is assured that provision of energy, protein, and micro-nutrients is adequate and that metabolic acidosis, hyperphosphataemia, and secondary hyperparathyroidism have been managed. This is fundamental for children with diminished height velocity and normal height SDS. An increase in resting energy expenditure has been described in children treated with rhGH, possibly related to increased protein turnover. Therefore, meticulous nutritional care should also be provided during the period of rhGH treatment. [82].

The positive effect of rhGH treatment on growth of dialysis patients is well documented. Fourteen patients on HD and 17 patients on PD were treated with rhGH for at least 12 months. The HD and PD patient groups were comparable with regard to age, bone age, height SDS, and height velocity at the start of treatment. During the first year of rhGH treatment, a nearly twofold increase in height velocity occurred in both groups. However, the efficacy of treatment decreased considerably during the second treatment year [83]. In addition, it is well documented that prepubertal growth acceleration with rhGH administration is not associated with a disproportionate advancement of bone age. These results were confirmed in a multicentre French study of 42 children on HD, with a height SDS below −2.0. During the first year of rhGH treatment there was a 0.5 mean SDS height gain [84]. Kari and Rees had similar results, in which the mean height SDS at the same period was increased by 0.4 [85]. Prepubertal children with CKD on conservative treatment respond better to rhGH than children on dialysis. In a German multicentre study it was found that the height gain of 41 patients on conservative treatment was 1.3 SDS, on average, during the first two treatment years compared with 0.8 SDS in 13 dialysis patients [15]. Despite evidence of rhGH efficacy and safety, the frequency of rhGH administration in children on dialysis remains low. This is partially explained by the lack of clear guidelines regarding the initiation and monitoring of GH therapy in children with CKD. Therefore, an algorithm has been developed recently for the rational evaluation and treatment of growth failure in this population by members of the consensus committee, outlining their recommendations for appropriate steps to improve growth [86].

The growth of children on dialysis has significantly improved during the past decade [10]. This is possibly the result of earlier referral, modern dialysis, appropriate nutritional management, the careful use of metabolites of vitamin D_3_, the use of recombinant human erythropoietin and, finally, the administration of recombinant human growth hormone. However, the growth of children 2–5 years of age remains a concern, and more aggressive nutritional management and wider use of recombinant human growth hormone might be required.
